# The Effects of Surface Gastrointestinal Electrical Stimulation on Gastrointestinal Function Recovery in Patients After Acute Type A Aortic Dissection Open-Heart Surgery: A Randomized, Controlled Trial

**DOI:** 10.31083/RCM39847

**Published:** 2025-10-31

**Authors:** Weitao Duan, Zeruxin Luo, Wei Huang, Xiu Zhang, Jianhua Su, Zhi Li, Miao Chen, Pengming Yu

**Affiliations:** ^1^Rehabilitation Medicine Center, Sichuan University West China Hospital, 610041 Chengdu, Sichuan, China; ^2^Department of Cardiovascular Surgery, Sichuan University West China Hospital, 610041 Chengdu, Sichuan, China; ^3^Key Laboratory of Rehabilitation Medicine in Sichuan Province, 610041 Chengdu, Sichuan, China

**Keywords:** rehabilitation medicine, cardiac surgery, electric stimulation therapy

## Abstract

**Background:**

Gastrointestinal (GI) dysfunction is a common postoperative complication in patients after acute type A aortic dissection (ATAAD) surgery. Recent evidence suggests that, in addition to early nutrition and feeding strategies, physiotherapy can help to reduce the incidence of postoperative GI dysfunction. This study aimed to investigate whether GI function after ATAAD open surgery can be recovered through surface gastrointestinal electrical stimulation (SGES).

**Methods:**

This was a prospective, parallel-group, assessor-blind, randomized controlled trial (RCT). A total of 74 participants were included and randomly divided into a control group (CG) and an SGES intervention group (IG) in a 1:1 ratio. The CG received a standardized perioperative management program developed by a multidisciplinary team, based on the principles of enhanced recovery after surgery (ERAS). The IG implemented SGES at ST36, ST25, and two additional GI pacemakers, as well as ERAS. The primary outcome was GI-2 recovery (tolerance of oral diet and passage of stool). Secondary outcomes included the Gastrointestinal Symptom Rating Scale (GSRS), acute gastrointestinal injury ultrasonography (AGIUS), the Gastrointestinal Quality of Life Index (GIQLI), the incidence of constipation and diarrhea, length of stay in the intensive care unit (ICU), and duration of hospitalization.

**Results:**

Of the 74 patients in this study, 24.32% were female, with a mean age of 49.61 years. The time to achieve GI-2 in the IG was significantly shorter, 1.9 days, than in the CG (log-rank test, *p* = 0.01). The GSRS scores in the IG were significantly lower than those in the CG (total scores: 1.2 vs. 1.6; *p* = 0.001). Moreover, the GIQLI values at all three follow-up visits were significantly higher in the IG group than in the CG group.

**Conclusions:**

To our knowledge, this is the first RCT to investigate the clinical effects of SGES on GI recovery after open-heart surgery for ATAAD. The results provide preliminary evidence supporting the feasibility and therapeutic potential of SGES in a high-risk population. SGES can promote the recovery of GI function, reduce GI-related symptoms, and improve the GI-related quality of life after open heart surgery in patients with ATAAD.

**Clinical Trial Registration:**

This trial was based on the Consolidated Standards of Reporting Trials (CONSORT) guidelines. This trial was registered in the Chinese Clinical Trial Registry (identifier ChiCTR2300075265, https://www.chictr.org.cn/showproj.html?proj=205523).

## 1. Introduction

Acute type A aortic dissection (ATAAD) is a rare but lethal cardiovascular disease, that accounts for approximately 60% of total aortic dissections (ADs) worldwide [1]. Patients who fail to receive prompt and comprehensive treatment in the early stage face a daunting mortality rate, estimated at 1% to 2% per hour [2], and a total mortality of 49% [3]. According to the international registry of acute aortic dissection (IRAD), surgical management can significantly decrease mortality from 23.7% to 4.4% at 48 hours, with a 3-year survival rate for discharged patients ranging from 69% to 90% [4]. Cardiac and aortic surgery remain the primary option and cornerstone of treatment for ATAAD patients [5]. However, patients who survive ATTAD surgery are commonly accompanied by long-term sequelae, including functional impairment and compromised quality of life. These are attributable to a spectrum of perioperative complications and diverse organ malperfusion [6, 7]. Furthermore, a retrospective study from China revealed the incidence of gastrointestinal (GI) dysfunction after ATAAD open surgery may reach 70%–80% if the occurrence of functional GI disorders, such as difficulty defecating, diarrhea, and abdominal distention, are considered [8, 9]. The occurrence of GI dysfunction has been shown to significantly increase the incidence of GI complications, prolong the intensive care unit (ICU) stay and duration of hospitalization after cardiac surgery [10, 11, 12]. Therefore, further improvement in the monitoring and management of GI function during the early postoperative phase is needed to reduce the incidence of GI dysfunction and facilitate the recovery of GI function in AD patients.

During the management of perioperative stage, the treatment of GI dysfunction in critically ill patients is mainly focused on early enteral nutrition, target-oriented fluid therapy, early transoral feeding, GI motility drugs, early physiotherapy and mobilization [13, 14, 15]. However, the clinical results of GI function recovery are still unsatisfactory, possibly due to the limitations of imposed by the early critical conditions of ATAAD patients.

Previous research has shown that transcutaneous electrical acupoint stimulation (TEAS) can improve postoperative GI dysfunction and reduce the duration of hospitalization [16]. Gastrointestinal electrical stimulation (GES) is another promising type of complementary therapy in GI dysfunction. In addition, GI pacing therapy is a type of electrotherapy based on the GI pacemaker theory. It generates a pacing current through the action of electrical pulses on the pacemaker area of the GI tract, thus regulating dynamic functions of the GI tract [17, 18]. Its therapeutic effects on the recovery of GI function include functional dyspepsia, abdominal distension, belching, anorexia, nausea, and gastroparesis, as well as intestinal dysfunction, irritable bowel syndrome, constipation, and other symptoms and diseases [19, 20].

Surface gastrointestinal electrical stimulation (SGES) is the combination of TEAS and GES. It may be a particularly promising option and clinical value in perioperative patients due to its non-invasiveness, low cost, and easy implementation compared with other invasive GI pacing therapies. Nevertheless, the application of SGES in patients after AD surgery is limited because of the pathophysiology of AD and dismal outcome of the AD population, and its clinical effects remain to be further clarified.

The principal objective of this study was to observe the efficacy of SGES in promoting GI function, reducing GI-related symptoms, shortening the duration of hospital stay, and improving the quality of life (QoL) of patients after AD surgery. This study is expected to provide evidence in favour of the application of SGES for perioperative patients, as well as a feasible pathway for the prevention and management of GI dysfunction in patients undergoing cardiac surgery.

## 2. Methods

### 2.1 Trial Design/Setting

This study was a prospective, single-centre, assessor-blind, parallel-group, randomized controlled trial (RCT). The clinical study protocol was structured according to the Consolidated Standards of Reporting Trials (CONSORT 2010) statement [21] and was conducted in compliance with the Declaration of Helsinki. The items of enrolment, intervention, and follow-up were coordinated and documented in accordance with the Standard Protocol Items: Recommendations for Interventional Trials 2013 (SPIRIT 2013) [22].

### 2.2 Study Participant 

This study was conducted concurrently within the Cardiovascular Thoracic Intensive Care Unit (CTICU) and Cardiovascular Surgery Ward, West China Hospital, Sichuan University (WCHSU). Eligible patients were randomly assigned to the intervention group (IG) or to the control group (CG). The recruitment of post-surgery patients with ATAAD began in September 2023 and ended in October 2024. Written informed consent (**Supplemental Material 1**) was obtained before discharge.

#### 2.2.1 Eligibility

Inclusion criteria:

∙ age 18–65 years;

∙ diagnosed with ATAAD and underwent open heart surgery.

Exclusion criteria:

∙ severe GI complications occurring at any time point after surgery including but not limited to those listed below: acute pancreatitis, ileus, GI bleeding, mesenteric ischemia, etc.;

∙ history of severe GI disease;

∙ any contraindications to conducting electrotherapy including but not limited to those listed below: pacemaker placement, impaired skin integrity, esthesiodermia, severe mental or psychological dysfunction, etc.;

∙ taking drugs before surgery that significantly affect GI function, including prokinetics or antidepressant drugs;

∙ IAP ≥20 mmHg;

∙ pregnancy at any time during the study;

∙ participation in other clinical trials previously [23].

Withdrawal criteria:

∙ unable to continue due to severe adverse events, or any other emergency issues requiring medical measures;

∙ any issue meeting the exclusion criteria;

∙ without compliance or being required to withdraw;

∙ any issue that violates the trial procedure [24].

The reasons for excluding and dropping out patients were recorded in detail in the case report form (CRF). Drop-out participants during intervention were mirror-replaced randomly by new participants to satisfy the requirements of this study.

#### 2.2.2 Trial Objectives

The primary objective of this trial was to explore the effects of SGES on GI electrophysiological changes in patients who undergo open heart surgery for ATAAD. The secondary objectives of this trial were to explore the effects of SGES on GI symptom scores, ultrasound scores, the incidence of GI dysfunction, QoL, and other health-related costs.

### 2.3 Randomization, Blinding, and Allocation

All eligible participants were randomly allocated into the CG or IG in a 1:1 ratio. The randomization program adopted a blocked randomization approach with a mixed block size of 2, 4, and 6 was employed to ensure baseline comparability between CG and IG. The sequence of block sizes was generated randomly by computer software to ensure unpredictability in group assignment. Each participant was assigned a computer-generated random three-digit number according to their sequence of enrolment. This number was placed in an opaque, tamper-evident sealed envelope, which was opened by an independent staff member who was not involved in recruitment or allocation. To further ensure the integrity of randomization, the staff member was blinded to the sequence and did not have access to any information about the group allocation before opening the envelope.

Given the inherent nature of this trial, it was impossible to blind the participants and interveners simultaneously. Therefore, we ensured that the assessor was blinded throughout the trial to mitigate the possibility of bias stemming from knowledge of the group allocation plan. Once the assessor was aware of their group information, another assessor was designated to conduct alternate assessments. Only the research director knew this allocation and coordinated the assessment and intervention independently. The intervener, assessor, and data processor were set separately.

### 2.4 Interventions

The timing of enrolment commenced within 24 hours of admission to the ICU after cardiac surgery. All eligible patients were offered a bundle of standardized perioperative management procedures based on the ICU multidisciplinary team, comprising intensive care specialists, critical care specialists, physiotherapists (PTs), respiratory therapists (RTs), and nutritionists. A list of comprehensive measures complied with the enhanced recovery protocol (ERP) mainly consists of early extubation, minimal sedation, optimal analgesia, reduction of surgical trauma, transoral feeding as soon as possible, early body mobilization, etc. The early progressive body mobilization program follows the ICU early mobilization protocol (‘Start To Move As Soon As Possible’ from UZ LEUVEN) [25], which is a validated and reliable early strategy for ICU patients. For the IG, the intervention involved standardized cardiac perioperative management procedures plus SGES, which uses a battery-powered portable device (KC-3000, Nanjing Kuan Cheng Technology Co., Ltd., Nanjing, China) and is registered as a class II medical device (REG.NO: SuzhouFDA20182260721). Regarding the parameters, the therapeutic current was pulse modulated intermediate frequency current (4 KHz carrier medium frequency plus 0.05 Hz–100 Hz low frequency wave), while the waveforms consisted of sine, square, stepped, exponential, triangular, trapezoid, sawtooth, and spike waves. The current intensity was individually tailored and determined as the maximum intensity (200 µA~1 mA) that produced “needling” or “massage-like” feelings that the patient can tolerate. An experienced PT implemented the intervention program and adjusted the voltage intensity according to the feedback of patients if necessary. Additionally, the given duration of treatment was 30 minutes once daily, initiating from the enrolment day until the day before discharge. Two output channels consisted of four selected therapeutic points. One output channel covered two GI pacemaker points, while the other channel covered two acupoints. Therapeutic point one (P1) was 1 cm above the umbilicus, while P2 was 5–10 cm to the right of the midpoint of the line between the xiphoid and the umbilicus. P3 and P4 were Zusanli (ST36) and Tianshu (ST25), respectively, which are both acupoints of the stomach meridian. P3 positioned at 3 cun below the right knee joint on the outer side of the lower limb, while P4 positioned at 2 cun right of the centre of the umbilicus. The size of the therapeutic electrodes ensured these areas were covered. For CG, standardized cardiac perioperative management procedures (ERP) were implemented without any other treatment.

### 2.5 Outcome Measures

Baseline measures, including demographics, surgery-related data, medical history, and comorbidities, etc., were collected at admission to the ICU within 24 hours.

#### 2.5.1 Primary Outcome

(I) GI-2. GI-2 was defined as the composite result of the time to tolerance of a transoral solid diet and the time to first defecation, which is a validated time indicator of recovery of GI function [26]. It has been proven that the composite parameters, GI-2, could better indicate the recovery of GI transit than the isolated time indicator (first flatus and first defecation, etc.) [27]. Related data were collected daily by an independent assessor and simultaneously referred to daily nursing records of the ICU to minimize potential bias.

#### 2.5.2 Secondary Outcomes

(II) Acute gastrointestinal injury ultrasonography (AGIUS). The AGIUS score is a bedside objective assessment used to monitor GI situations in critical care population; in particular, it has predictive value for assessing feeding intolerance and for guiding enteral nutrition protocols [28]. Two well-trained medical staff members performed ultrasonography examinations (curvilinear probe, 2–5 MHz, Nanjing, China). The standard program of the AGIUS was used twice separately: in the afternoon after admission to the ICU, and the day before discharge [29]. The examination consisted of the diameter of the intestine, the thickness of the intestine, and intestinal peristalsis, which contributes to identifying early recovery of GI function and other GI complications. Final AGIUS scores were collected from the electronic records of the ultrasonic machine.

(III) Intra-abdominal pressure (IAP), is also a basic indicator of GI structural recovery and is often used to monitor abdominal conditions. In this study, bladder pressure was used to represent internal abdominal pressure. The standard detection method was as follows: in the case of urinary tube insertion, subjects were completely supine, the abdomen was completely relaxed at the end of exhalation, and no more than 25 mL normal saline was injected into the bladder at the midaxillary line level as zero, and the readings were taken at the end of exhalation [30]. The record unit was mmHg, and IAP were acquired twice by experienced ICU nurses at the admission to ICU and after ICU.

(IV) Incidence of constipation and diarrhea. Constipation was defined as the absence of bowel movement (BM) for three days after oral intake. Diarrhea was defined as having more than three BMs in a single day or a total stool volume exceeding 750 mL [31]. The events of defecate were documented from the daily nursing records of the ICU.

(V) Gastrointestinal Symptom Rating Scale (GSRS) is commonly used to assess functional GI disorders, perioperative GI dysfunction, or critical care units and has well-documented reliability and validity [32]. The GSRS consists of 5 GI symptom clusters and 15 items (including abdominal pain, constipation, reflux, etc.) and is scored on a 7-point Likert scale to indicate the severity of each symptom. The GSRS was collected at the discharge morning based on the symptoms recalled during the hospital stay [33].

(VI) Gastrointestinal quality of life index (GIQLI) has been reported to be a validated and convenient tool for evaluating long-term GI-related symptoms and QoL. It includes 5 dimensions (symptoms, emotions, physical and social function, and treatment impact) and 36 items on a 5-point Likert scale, with total scores ranging from 0–144 [34]. This scale was used for follow-up visits, and all of the subitems and total scores in the CRF were recorded.

(VII) Health-related costs included the length of ICU stay and the duration of hospitalization.

### 2.6 Sample Size Calculation

Sample size calculations were based on the change in GI-2 between IG and CG. Due to the lack of reported research data concerning the effects of SGES on GI function after AATAD surgery, we referred mainly to the results of a preliminary experiment. According to the results of the preliminary experiment, the hazard ratio (HR) for GI-2 after SGES was 1.8. The sample size was determined by G*POWER V3.1 with a test power of 0.8 (β = 0.20) and a significance level of α = 0.05 (two-sided test). The numbers of patients in both groups was equal, and it was calculated that each group required 37 patients. Considering the high dropout rate of AATAD patients (20%), 45 patients needed to be included in each group, and a total of 90 patients needed to be included in the two groups.

### 2.7 Statistical Analysis

Two statisticians independently performed the statistical analysis via SPSS version 26.0 software (IBM Corp., Armonk, NY, USA). If missing values were present, multiple imputation of missing data was performed (R x64 4.0.4 R Foundation for Statistical Computing, Vienna, Austria). Sensitivity analysis (per-protocol) was performed to compare the variability of results between filled and unfilled data. Continuous variables with a normal distribution were described using the mean and standard deviation (SD), and Student’s *t* test was used to determine differences between IG and CG. Non-normally distributed quantitative data was described by the median and range, and qualitative variables were described by numbers and proportions (%). The Mann‒Whitney *U* test was used for nonnormally distributed data. Categorical variables were tested via χ^2^ test or Fisher’s exact tests (applied when more than 20% of the expected frequencies in the contingency table were less than 5). For ordered categorical variables, the Cochran-Armitage trend test was used to evaluate linear trends across groups, when applicable. The significance level was set at *p* < 0.05. Kaplan–Meier curves were constructed for the primary outcome.

## 3. Results 

### 3.1 Participant Characteristics

**Fig. 1.**
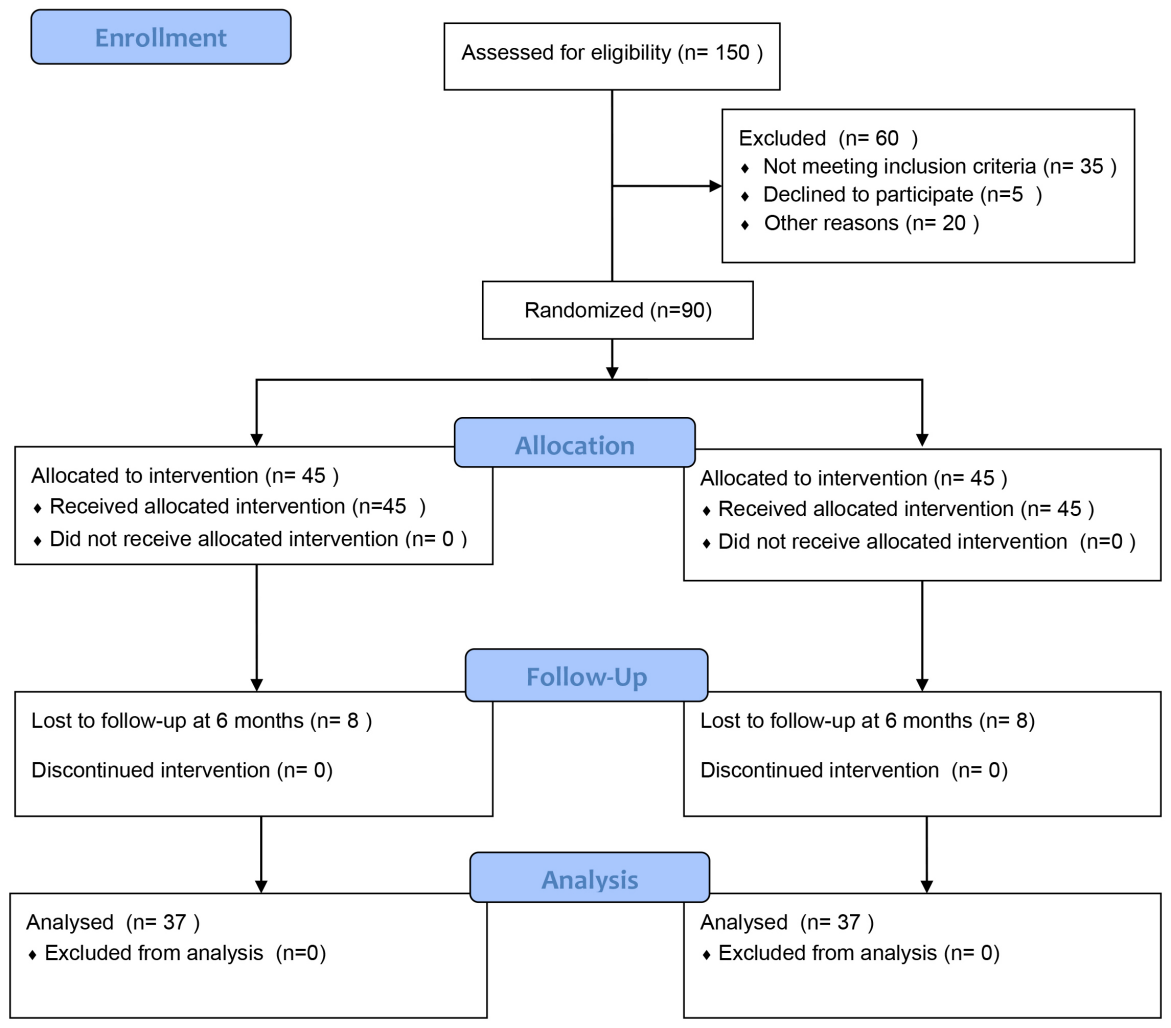
**Consolidated standards of reporting trials (CONSORT) diagram**.

The recruitment of this trial is summarized in the following CONSORT flowchart below (Fig. 1). From September 2023 to October 2024, 150 ATAAD patients underwent open heart surgery were recruited by the Cardiovascular Thoracic Intensive Care Unit (CTICU) at the West China Hospital, Sichuan University (Fig. 1). A total of 60 patients were excluded from the study (35 for not meeting inclusion criteria, 5 for refusing to participate, and 20 for other reasons). 90 patients were randomly assigned to the two groups (45 in each). 8 patients were lost to follow-up in each of IG and CG, and 37 patients in each group were included in the final analysis.

Of the 74 randomized patients in the final study cohort, 24.32% were female, and the mean age was 49.61 years. The mean duration of cardiopulmonary bypass was 177.68 min, and the IAP of at admission to ICU was 9.5 cmH_2_O. The primary baseline characteristics of participants are shown in Table 1.

**Table 1.** **Baseline demographics and clinical characteristics of the study population**.

		IG (n = 37)	CG (n = 37)	*p* value
Female, n (%)		9 (24)	9 (24)	1.000
Age (yrs), mean (SD)		48 (9)	51 (12)	0.222
Height (cm), mean (SD)		168.5 (8.6)	168.3 (9.8)	0.940
Weight (kg), mean (SD)		75.2 (15.1)	70.6 (14.0)	0.178
BMI, mean (SD)		26.3 (3.9)	24.8 (3.5)	0.076
History of smoke				0.888
	Non-smoker, n (%)	21 (57)	21 (57)	
	Smoker, n (%)	13 (35)	14 (38)	
	Ex-smoker, n (%)	3 (8)	2 (5)	
History of drink				0.668
	Non-drinker, n (%)	25 (68)	23 (62)	
	Drinker, n (%)	10 (27)	13 (35)	0.668
	Ex-drinker, n (%)	2 (5)	1 (3)	
Characteristics of surgery				
	Duration of operation (min), mean (SD)	406 (81)	419 (75)	0.464
	Duration of cardiopulmonary bypass (min), mean (SD)	182 (46)	173 (30)	0.291
	Duration of anesthesia (h), mean (SD)	8.5 (1.5)	8.7 (1.1)	0.618
	Emergency operation, n (%)	18 (49)	18 (49)	1.000
	IAP (cmH_2_O), mean (SD)	9.5 (2.4)	9.5 (3.3)	0.968
Comorbidities, n (%)				
	Hypertension	34 (92)	31 (84)	0.286
	Diabetes mellitus	2 (5)	4 (11)	0.394
	Hyperlipidemia	1 (3)	0 (0)	0.314
	Cardiovascular disease	4 (11)	5 (14)	1.000

Data are presented as number, percentage (%) and mean (SD). IAP, Intra-abdominal pressure; CG, control group; IG, intervention group; BMI, body mass index; yrs, years; min, minutes; h, hours. The Chi square test, Student’s *t*-test and Mann-Whitney *U* test were applied to compare between groups at baseline.

### 3.2 Primary Outcomes

#### Comparison of GI-2 Between IG and CG

**Fig. 2.**
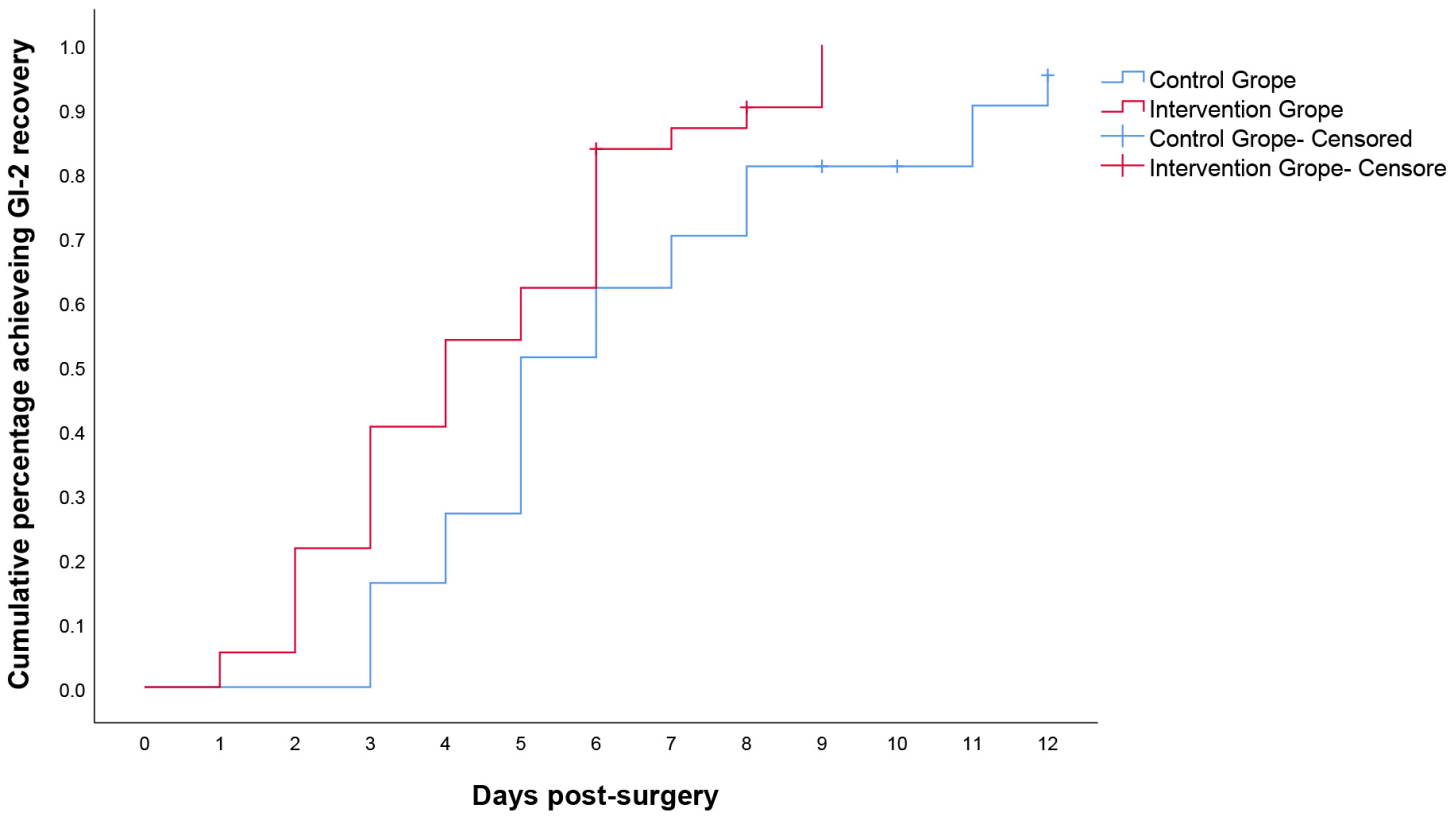
**Kaplan–Meier curves for time to achieve GI-2**. GI-2, validated composite measure was defined as the interval from surgery until the first passage of stool and tolerance of solid intake for 24 h in the absence of vomiting. *p* = 0.01 (log rank test, χ^2^ = 6.620).

Overall, 68 of the 74 patients (91.9%) achieved GI-2, 35 (94.6%) in IG and 33 (89.2%) in CG. GI-2 was 1.9 days (4.5 (95% confidential interval (CI): 3.8–5.3) vs 6.4 (95% CI: 5.4–7.3)) shorter in IG than in CG with a median of 4 days (95% CI: 2.5–5.5) vs 5 days (95% CI: 4.0–5.9); *p* = 0.01 (Fig. 2). Six patients (8.1%) were censored for the GI-2 recovery end point because their first flatus or toleration of clear liquids was considered adequate for discharge by the investigators rather than toleration of solid food or BM.

### 3.3 Secondary Outcomes

#### 3.3.1 Comparison of AGIUS Between IG and CG 

No statistical difference in AGIUS was observer between IG and CG upon admission to ICU (0.78 vs 0.85, *p* = 0.232) and at discharge (0.49 vs 0.45, *p* = 0.723). However, statistical differences were observed for two measurements with the AGIUS scores in both groups decreasing over time. There were no statistical differences in AGIUS sub-scores, except for intestinal peristalsis at discharge, with IG showing better intestinal peristalsis than CG (0.81 vs 0.54, *p* = 0.034) (Table 2).

**Table 2.** **Acute gastrointestinal injury ultrasonography scores for IG and CG**.

AGIUS (SD)	Admission to ICU		*p* value	*t*	Discharge from ICU		*p* value	*t*
	CG	IG			CG	IG		
Diameter of intestine	0.41 (0.49)	0.42 (0.50)	0.490	0.694	0.32 (0.48)	0.22 (0.42)	0.302	1.041
Thickness of intestine	0.70 (0.46)	0.62 (0.49)	0.468	0.730	0.41 (0.50)	0.43 (0.50)	0.817	0.232
Intestinal peristalsis	1.32 (0.58)	1.35 (0.48)	0.828	0.218	0.81 (0.52)	0.54 (0.56)	0.034	1.011
Total score	0.85 (0.23)	0.78 (0.25)	0.232	1.204	0.49 (0.21)	0.45 (0.21)	0.823	0.225

Data are presented as the mean value. AGIUS, acute gastrointestinal injury ultrasonography. Calculated used by Student’s *t*-test.

#### 3.3.2 Comparison of the Incidence of Constipation and Diarrhea Between IG and CG

There was no significant difference in the incidence of constipation and diarrhea between the two groups during hospitalization based on previous definition. The incidence of constipation was 54.1% in IG vs 59.5% in CG, (*p* = 0.639); the incidence of diarrhea was 27.0% in IG and 21.6% in CG, (*p* = 0.588) (Table 3).

**Table 3.** **Comparison of the incidence of constipation and diarrhea between IG and CG**.

	CG	IG	χ ^2^	*p* value
The incidence of constipation	22 (59.5)	20 (54.1)	0.220	0.639
The incidence of diarrhea	10 (27.0)	8 (21.6)	0.294	0.588
The incidence of constipation and diarrhea	28 (75.7)	25 (67.6)	0.598	0.439

Data are presented as the number and percentage. Statistically analysis was calculated by the Chi-Square Test.

#### 3.3.3 IAP After ICU, and IAP Changes Within CG and IG

No significant differences were observed between CG and IG both in IAP after ICU (8.5 vs 8.7, *p* = 0.590) and IAP change within groups (Table 4).

**Table 4.** **IAP after ICU, and IAP changes within CG and IG**.

	CG	IG	*t*	*p* value
IAP after ICU	8.5 (2.5)	8.7 (2.1)	–0.361	0.590
IAP change within CG	0.96 (3.2)	-	1.805	0.079
IAP change within IG	-	0.79 (2.6)	1.841	0.074

Data are presented as the mean value and SD in cmH_2_O. IAP change, the difference value between admission and after ICU within groups; Statistically analysis was calculated by the paired *t*-test.

#### 3.3.4 Comparison of GSRS Between CG and IG

Total symptom scores (TSS) of GSRS were significantly lower in IG than CG (1.2 vs 1.6, *p* = 0.001). Although not stratified by individual symptoms, the greatest improvement in TSS was observed in the indigestion and reflux sub-scores. Other sub-scores such as constipation and diarrhea were also lower in IG than in CG, but the differences were not statistically significant (Table 5).

**Table 5.** **Gastrointestinal symptom rating scale of CG and IG**.

	CG (SD)	IG (SD)	*t*	*p* value
Abdominal pain	1.5 (0.6)	1.2 (0.3)	2.605	0.011
Indigestion	1.5 (0.5)	1.3 (0.4)	2.338	0.022
Reflux	1.1 (0.4)	1.2 (0.5)	–0.525	0.601
Constipation	2.1 (1.1)	1.6 (0.8)	2.000	0.049
Diarrhoea	1.6 (0.9)	1.5 (0.7)	0.700	0.486
TSS	1.6 (0.3)	1.2 (0.2)	3.600	0.001

Date are expressed as the mean value and SD; TSS, total symptom scores. Statistically analysis was calculated by the Student’s *t*-test.

#### 3.3.5 Comparison of GIQLI Between CG and IG

**Fig. 3.**
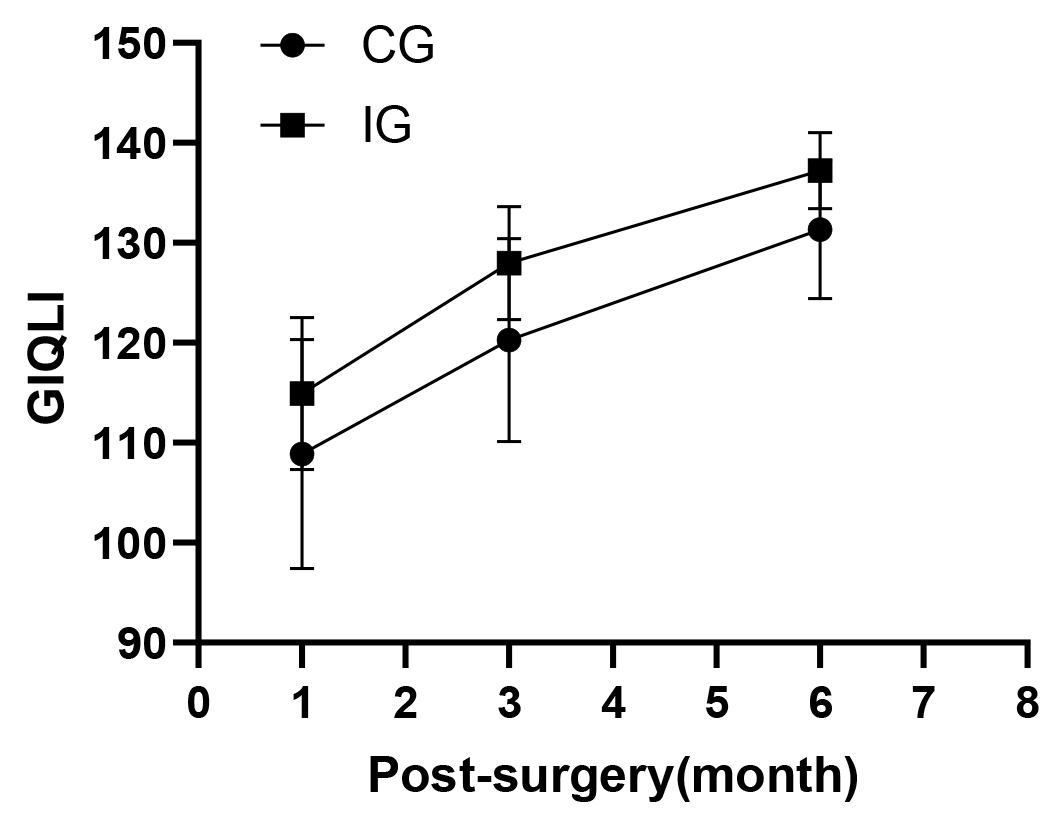
**Gastrointestinal system quality of life index scores**. GIQLI, Gastrointestinal quality of life index.

There were statistically significant differences in GIQLI between the IG and CG at all three follow-up time points as evidenced by the nearly parallel curves in Fig. 3. The GIQLI scores in IG were higher than those of CG (TI: 115 vs 109; T2: 128 vs 120; T3: 137 vs 131). There were also differences at all three time points within IG and CG. The GIQLI scores increased over time in both groups, but only two patients reported 100% recovery in the 6th months after discharge. IG showed better recovery in GIQLI over the three follow-up compared to CG (Table 6).

**Table 6.** **Gastrointestinal system quality of life index scores of CG and IG**.

GIQLI (M ± SD)	1 month follow-up	3 month follow-up	6 month follow-up	*F* test		
				*F*	*p*	η ^2^
CG	109 (11)	120 (10)	131 (7)	-	-	-
IG	115 (8)	127 (6)	137 (4)	-	-	-
Group Main Effect	-	-	-	14.444	<0.001	0.997
Time Main Effect	-	-	-	648.887	<0.001	0.900
Group Time Interaction	-	-	-	1.294	0.274	0.018

Data are presented as the mean value and SD. Statistical analysis was calculated by Repeated Measures ANOVA.

#### 3.3.6 Comparison of Health-related Costs Between CG and IG

Overall, the mean ICU stay and duration of hospitalization were 3.8 days (95% CI: 3.1–4.5 and 11.8 days (95% CI: 10.7–13.0), respectively. The length of ICU stay was 3.8 days (95% CI: 3.1–4.5) in IG and 4.1 days (95% CI: 3.5–4.6) in CG, with no significant difference between the two groups. The duration of hospitalization was 11.8 days (95% CI: 10.7–13.0) in IG and 13.3 days (95% CI: 11.4–15.2) in CG, again with no significant difference between the two groups (Table 7).

**Table 7.** **Health-related costs between the two groups**.

	CG	IG	*t*	*p* value
Length of ICU stay (days)	4.1 (95% CI 3.5–4.6)	3.8 (95% CI 3.1–4.5)	0.634	0.528
duration of hospitalization (days)	13.3 (95% CI 11.4–15.2)	11.8 (95% CI 10.7–13.0)	1.323	0.190

Date are expressed as the mean value and 95% confidential interval (CI). Statistically analysis was performed with Student’s *t*-test.

## 4. Discussion

The results of this study provide novel evidence regarding the effects of SGES based on ERP on GI function recovery after ATAAD open-heart surgery. This approach achieved the promotion of GI-2, that is, earlier postoperative transoral feeding and defecation, reduced GI-related symptom scores, and improved long-term GI-related QoL index.

Despite these encouraging findings, it is important to interpret the results in the context of the complex clinical landscape of ATAAD patients. GI recovery in this population is often complicated by severe physiological stress, surgical trauma, and heterogeneous postoperative trajectories. While SGES showed benefits in certain surrogate outcomes such as GI-2 and GSRS, no statistically significant effects were observed in more difficult to achieve endpoints such as constipation, diarrhea, IAP, or the duration of ICU/hospital stay. These results may reflect both the multifactorial nature of GI dysfunction and the possibility that TES offers modest, supportive effects rather than transformative clinical impact in this setting.

Recently, standardized enhanced perioperative management protocols have been introduced to facilitate GI recovery following major surgeries [35, 36]. However, patients undergoing cardiac surgery are prone to experience GI dysfunction, mainly because of reduced GI blood supply due to surgical trauma, stress reactions, and poor preoperative perfusion, all of which can negatively affect prognosis [37]. Although numerous RCTs have explored the clinical efficacy of various gastrointestinal electrical stimulation (GES) methods for general GI dysfunctions such as constipation and functional dyspepsia, their therapeutic potential and clinical applicability following open-heart surgery for ATAAD have yet to be investigated [38]. This gap in research may be attributed to the significant heterogeneity among ATAAD patients, the critical condition of these patients in the early postoperative stage, and the challenges associated with patient recruitment and long-term follow-up. The definition of GI dysfunction remains ambiguous, and there is currently no standardized approach for assessing and determining the extent of GI recovery [39]. Given that time to first flatus is considered an unreliable measurement, the current study employed the objective, composite time-based indicator GI-2 [40]. This was complemented by ultrasound assessment and GI symptom scores to evaluate multiple aspects of recovery and to enhance the sensitivity of assessment. We believe the development of such a comprehensive evaluation system for GI function holds promising clinical applicability and may improve the accuracy and consistency of postoperative assessments.

In this prospective RCT, SGES shortened the time to GI function recovery (GI-2) after open-heart surgery for ATAAD by 1.9 days compared to CG. This finding, is similar to the previous results for abdominal surgery [41]. Previous study showed that GES significantly improved GIQLI scores and symptom scores [42]. Consistent with this, in the present study SGES significantly reduced the total postoperative GI-related symptom scores during hospitalization as well as showing benefits in long-term GIQLI during follow-up. Although these improvements did not translate into significant differences in the incidence of constipation or diarrhea, AGIUS scores, length of ICU stay, or duration of hospitalization the study may have been underpowered to detect such effects.

No statistically significant differences in AGIUS results were found between IG and CG, although IG showed a improvement in intestinal motility as detected by ultrasound compared to CG. The relationship between intestinal ultrasound and gastrointestinal recovery is still unclear [43]. The primary outcome measures of interest in this study may not have been fully captured due to the complex pathophysiology and mechanism of postoperative GI dysfunction [44]. No previous study reported that GES can improve IAP. Similarly, there were no significant differences in the IAP measurement or IAP changes between IG and CG. Additionally, both the AGIUS and IAP measurements were relatively low in both groups, indicating that none of the enrolled patients had significant organic GI disorders. This aligns with the inclusion criteria for our study, which excluded patients with severe organic GI disease or high IAP in order to ensure baseline consistency.

The results of GSRS and GIQLI revealed that IG experienced significant positive effects in both short-term (assessed within one week of hospitalization) and long-term (assessed during the two weeks post-discharge) GI-related QoL. These improvements were observed for both early postoperative recovery and long-term recovery, thus highlighting the positive effects of SGES on GI-related QoL. The findings also indicated that the majority of patients undergoing ATAAD surgery had not fully regained 100% GI function by six months postoperatively. A previous study reported similar findings, with the health-related QoL of ATAAD patients showing a significant long-term decline after surgery [45]. This outcome may be due to multiple factors, including disease pathophysiology, surgical trauma, the complex effects of medication, reduced physical activity, and psychological or emotional influences [46].

The stimulation parameters and treatment area greatly influence the mechanism of action of GES [47]. The sites selected for electrical stimulation in this study were the projection of GI pacemaker on the body surface and two main acupoints. According to a previous systematic review and meta-analysis, ST36 and ST25 are the most commonly selected acupoint for transcutaneous electrical acupoint stimulation (TEAS), that can improve postoperative GI dysfunction [48]. Projection of the GI pacemaker covered by the electrode on the body surface by modulated medium frequency electrotherapy may have similar therapeutic effects, and is different to invasive GI pacemaker electrical stimulation [49]. Similar to interferential current (IFC), the current frequency (4 kHz plus 0.05 Hz–100 Hz) selected in this study is better tolerated and well suited to penetrate deeper tissues for GI dysfunction [50, 51]. Moreover, although SGES incorporates acupoint stimulation and noninvasive GI pacing, the precise biological mechanisms underlying its effects remain incompletely understood. Hypotheses include neuromodulation of autonomic control, enhancement of GI pacemaker activity, and reduction of inflammation. However, direct mechanistic evidence in post-cardiac surgery patients is still lacking.

Of note, the adoption of TES in clinical practice remains limited, especially in cardiac surgery patients. This is due to concerns surrounding feasibility, cost-effectiveness, and the need for standardization. Our study does not seek to recommend routine TES use, but rather seeks to stimulate further inquiry. We consider this work to be hypothesis-generating and exploratory, with the goal of opening new avenues for multidisciplinary interventions that target GI recovery in high-risk surgical patients.

## 5. Limitations

This study has several limitations. It was conducted in a medical center with a strict ERP, which may limit the generalizability of the results to other settings. Due to the nature of the intervention, it was deemed impractical to implement blinding for both the participants and assessors. However, the blinding principles were strictly followed for data assessors, with independent arrangements for the interventionists, assessors, and data handlers. Data analysis was also conducted under blinded conditions with respect to group allocation. The absence of a placebo-controlled group in this trial means that the placebo effect could not be excluded, which may impact the interpretation of treatment outcomes. Additionally, challenges with patient recruitment resulted in a smaller sample size and a higher than expected dropout rate during follow-up. These issues were primarily due to the geographical distribution of some patients in remote areas of China. Another noticeable consideration is that while an attempted was made to address the significant heterogeneity among TAAD patients by ensuring consistency in baseline surgical data (e.g., duration of surgery, extracorporeal circulation time, and anesthesia time), the potential influence of variations in surgical techniques on the study outcomes remains uncertain. Furthermore, the physiological basis for SGES remains speculative, with no direct biomarkers or electrophysiological endpoints included. Finally, this study reflects an early-stage clinical trial, and we caution against interpreting the results as conclusive.

If possible, future research should focus on a specific type of surgical approach to minimize variability and improve the precision of results. It may also be worthwhile to consider whether ultrasound and IAP measurement should be used as routine monitoring tools for GI dysfunction after ATAAD open heart surgery. Future large-scale clinical studies are expected to further reveal the optimal therapeutic parameters and mechanisms of SGES therapy. We recommend further multicenter trials with a larger sample size, standardized stimulation protocols, and embedded mechanistic assessments to further evaluate TES. SGES may represent a feasible, non-invasive adjunctive strategy, but its role must be more clearly defined through rigorously designed studies.

## 6. Conclusions

This study provides preliminary evidence supporting the clinical benefits of SGES in improving GI function and QoL, as well as the reduction of GI symptoms scores after open heart surgery in ATAAD patients. Further large-scale studies are warranted to confirm these findings and to explore the broader implications and mechanisms of SGES in postoperative recovery strategies for cardiac surgery patients.

## Availability of Data and Materials

The datasets used and analyzed during the current study are available from the corresponding author on reasonable request.

## Supplementary Material

Supplementary material associated with this article can be found, in the online version, at https://doi.org/10.31083/RCM39847.
