# Supplementary material for: The Effects of Surface Gastrointestinal Electrical Stimulation on Gastrointestinal Function Recovery in Patients After Acute Type A Aortic Dissection Open-Heart Surgery: A Randomized, Controlled Trial
Source: Rev Cardiovasc Med. 2025 Oct 31;26(10):39847. doi: 10.31083/RCM39847 (PMC12593729; doi:10.31083/RCM39847)
Supplement: Supplementary file 1 [file 2153-8174-26-10-39847-s1.zip › Supplemental Material 1.docx]

**Informed Consent Form**

**Dear participant:**

You are invited to participate in a study approved by the West China Hospital in Sichuan Province on the study for investigating the effects of **surface gastrointestinal electrical stimulation (SGES)** on gastrointestinal function recovery in patients after acute Type A aortic dissection open heart surgery. An estimated 90 subjects will volunteer to participate in this study. The study has been reviewed and approved by the Biomedical Ethics Review Committee of the West China Hospital of Sichuan University.

**1. Why is this study being undertaken?**

Patients who survive acute Type A aortic dissection(ATAAD) open heart surgery are commonly accompanied by long-term sequelae, including functional impairment and compromised quality of life, attributable to a spectrum of perioperative complications and diverse organ malperfusion. Gastrointestinal (GI) complications are uncommon but life-threatening complications after cardiac surgery, particularly in the context of ATAAD. Moreover, early GI dysfunction emerges as an important risk factor for a high incidence of GI complications and subsequent mortality post-cardiac surgery. Therefore, it is necessary to further strengthen the monitoring and management of GI function during the early postoperative phase for AD patients to reduce the incidence of GI complications and facilitate the recovery of GI function.

GI dysfunction management and treatment in critically ill patients mainly focus on early enteral nutrition, target-oriented fluid therapy, early transoral feeding, GI motility drugs, early physiotherapy and mobilization, as well as modalities like acupuncture or acupuncture-like and electrical stimulation therapy, etc. The effects of GI pacing therapy on the recovery of GI function include functional dyspepsia, abdominal distension, belching, anorexia, nausea, gastroparesis, as well as intestinal dysfunction, irritable bowel syndrome, constipation, and other symptoms and diseases, which have been widely verified. In addition, SGES presents a particularly promising option and application value in perioperative patients due to its non-invasive, low-cost, and easy implementation.

The principal objective of this study is to observe the efficacy of SGES in promoting GI function, reducing the incidence of GI complications, shortening hospital stay duration and improving quality of life in patients after AD surgery.

**2.What do you need to do if you take part in the study?**

You will be randomly allocated to the intervention group and the control group. Post-operative SGET will be conducted from the second after admission to the ICU before discharge. In addition, you will be required to come to the hospital for several questionnaires at the 1st, 3th and 6th month after surgery.

**3.What are the treatment options available?**

If you are unable or unwilling to participate in this study, you still will receive standard perioperative management based on ERAS principal which will include proper rehabilitation intervention and health instructions. After discharge, you will not be guaranteed to make an appointment with a professional physiotherapist, which can be inconvenient and costly in terms of energy and money.

**4. Who should not take part in the study?**

If you are a patient with severe gastrointestinal complications, history of severe gastrointestinal disease, any contraindications to conducting electrotherapy like pacemaker placement, pregnancy, taking drugs significantly affecting gastrointestinal function should not be included in this study.

**5.What are the risks of participating in the study?**

In this study, SGES as a kind of body surface electrical stimulation, may occasionally induce adverse events including GI symptoms and local skin reactions such as abdominal pain, diarrhoea, local skin allergies, redness, as well as mild discomfort at the treatment site. All of the potential adverse reactions could be completely recovered upon discontinuation of therapy. Once adverse events occur, regardless of severity, will immediately inform the emergency team to determine the corresponding medical measures and simultaneously report to the safety management department and manufacturer to assess the causality of adverse events.

**6. What are the possible benefits of taking part in the study?**

Taking part in this study will help you recover fast after surgery and improve your gastrointestinal function, mitigate your gastrointestinal symptoms and promote early transoral solid diet and defecation, and improve quality of life. The study will also help determine whether BGIT is a beneficial option for gastrointestinal function recovery and reduce the cost of registration fees due to billing for tests.

**7. Do I need to pay for my participation in the study?**

There is no charge for assessment and intervention in this study and you do not need to register for the process.

**8. Is personal information confidential?**

Your research data will be kept at West China Hospital of Sichuan University and your medical records will be accessible to the investigator, the research authority, and the ethics review committee. Any public reports of the results of this study will not disclose your personal identity. We will make every effort to protect the privacy of your personal medical information and personal information to the extent permitted by law.

**9. Do I have to take part in the study?**

Participation in this study is completely voluntary and you may refuse to participate in the study or withdraw from the study at any stage of the trial at any time without discrimination or reprisal and without prejudice to your medical treatment and rights. If you decide to withdraw from the study, please contact your doctor for proper treatment of your illness.

Subject declares that I have read the above description of this study and that my researcher has fully explained and justified to me the purpose, the operational procedures and the possible risks and potential benefits of participating in this study, and has answered all my relevant questions. I voluntarily agree to participate in this study.

I agree□ or refuse□ to use my research data and biological specimens in addition to this research.

Printed Name of Participant: ＿＿＿＿＿＿＿＿＿＿＿＿

Signature of Participant: ＿＿＿＿＿＿＿＿＿＿＿＿

Date: ＿＿＿＿＿＿＿＿＿＿＿＿＿

Phone of Participant: ＿＿＿＿＿＿＿＿＿＿＿＿

Printed Name of Legal representative: ＿＿＿＿＿＿＿＿＿＿＿＿ (If applicable)

Relationship with subjects: ＿＿＿＿＿＿＿＿＿＿＿＿

Signature of Legal representative: ＿＿＿＿＿＿＿＿＿＿＿＿

Date: ＿＿＿＿＿＿＿＿＿＿＿＿＿

Reason for signing by legal representative: ＿＿＿＿＿＿＿＿＿＿＿＿

Printed Name of Witness: ＿＿＿＿＿＿＿＿＿＿＿＿ (If applicable)

Signature of Witness: ＿＿＿＿＿＿＿＿＿＿＿＿

Date: ＿＿＿＿＿＿＿＿＿＿＿＿＿

Reason for signing by Witness: ＿＿＿＿＿＿＿＿＿＿＿＿

Declaration by the doctor: I have explained the relevant details of this study to the

above-mentioned volunteer participating in this study and have provided him/her with a

signed original copy of the informed consent form. I confirm that I have explained in

detail to the subject the study, in particular the ethical principles and requirements of

possible risks and benefits, free of charge and compensation, damages and damages,

voluntariness and confidentiality that may arise from participation in this study.

Doctor's signature:______________

Date:__________

Doctor's phone number:__________

Biomedical Ethics Review Committee of West China Hospital, Sichuan University
